# Chicago Society of Physicians and Surgeons

**Published:** 1874-11

**Authors:** Ralph E. Starkweather


***Chicago Society of Physicians and Surgeons.*** *Transactions at Special Meeting, September* 2, 1874. Reported by RALPH E. STARKWEATHER, M.D.

The President, Dr. John Bartlett, in the Chair. The meeting had been called, by request of several members, in order that the Society might hear Dr. Ely McClellan, Assistant Surgeon of the U. S. Army, speak informally upon the cholera epidemic of 1873. He said that in 1873 Pelikan and other Russian sanitarians startled the medical world by declaring that cholera had become endemic in Russia. Recently, Dr. Radcliffe, at a meeting of the British Medical Association, demonstrated the error of this conclusion. He then pointed out upon the map the two lines of travel from India to Europe, the North Persian, the route by way of Jelalabad, Herut, Meschid to Asterabad upon the Caspian sea, across to Baku, thence overland to Tiflis and to Poti, upon the Black sea. Secondly, the Red Sea route, from India, by way of Bombay, down the river Indus to Kurrachee, thence up the Persian gulf, via Shiraz and Ispahan, to Teheran, the junction of the north and south Persian routes. Kiev, the holy city of Russia, is located on the river Dnieper, and is visited annually by thousands of pilgrims. In 1869 the cholera spread from Kiev down the river Dnieper to Odessa. It arrived 1870 on the Baltic sea, by the rivers Niemen and Vistula, which are connected with the Dnieper by a series of canals. In 1871, cholera prevailed in Prussia and Austria; in 1872, Austria, Prussia, Italy, France and Holland; in 1873, in Great Britain. In 1873, too, cholera entered New Orleans, from Europe: no less than seventy-three vessels having arrived from infected ports.

The history of an initial case was then given with great clearness and rapidity, of an Ohioan sick with the cholera on a river steamer from New Orleans. He died before reaching Memphis, at which place his remains, as well as his garments and soiled clothes, were taken ashore, and the body put into a casket and forwarded to his home. The German woman who washed his garments and his body, died from cholera two days afterwards. From these two cases the epidemic originated, and from none other. Near this city various public works were in progress, canals and railroads. It was in this city (Memphis) that the great outbreak or explosion of cholera took place—(it was not the first point of infection, however)—from which in all directions the disease was carried. The various lines of railroad in the State of Kentucky were pointed out as having numerous towns into which cholera had been brought by commercial travelers, by negroes, or by infected clothing. Then various counties of Kentucky were mentioned as having peculiarities of the epidemic, due to their remoteness from lines of travel, or to streams of water, or good general hygienic condition. It was in Adair county that the cholera was most malignant of the counties of that State—Kentucky—especially in the town of Columbia, where in the rear of a livery stable there was a privy vault full of the most foul putridity; the ground adjoining it was saturated with the fluid from this pest-hole. The proprietor of the stable rejected all attempts made to have it cleaned. Late in August, a negro who had cholera used this privy; from this point of origin, twenty-six persons lost their lives from cholera. Reference was then made to the Marion County Fair—Kentucky—the doctor explaining how universal is the custom among the people, of attending these gatherings; and said that the fair held in August of 1873 proved a most disastrous means of general dissemination of cholera through that and all the adjacent counties. He was understood to say that it was in one of the patients who took cholera at this time and place, that there was *complete retention of urine for five and one-half days, with subsequent recovery.*

The depressing influences of a panic were forcibly and vividly illustrated by the cases in Garrard county, Ky. In 1836, there had been a devastating epidemic of cholera in that county, and from that time the people had handed down stories of its ravages and sorrowful scenes. When, therefore, after combating the disease in 1873 as being cholera morbus, it was declared to be Asiatic cholera, the consternation and panic became overwhelming, and the disease very virulent and fatal.

A graceful tribute was paid to the authorities of Lancaster, Garrard county, Ky., for that which had seldom been done elsewhere, namely, paying the physicians who labored to subdue the disease; they also disinfected the districts, and took care of the poor, at the public expense.

Dr. McClellan said that he was anxious to obtain the records of the epidemic in the direct line of the disease; he had no desire to be *ex parte*, or to support any theories, but simply to secure facts. He wished physicians would record their cases, and would be glad if they would give him notes of any, stating the details as minutely as possible, such as the sex, age, color, condition in life, date of attack, date of recovery, date of death, remarks, and treatment.

As to the pathology and treatment of cholera, little was said, for lack of time. It was considered to have three stages. The first, was the premonitory painless diarrhœa. Cholera is the only malignant disease amenable to treatment in its first stage; this should be promptly treated, else, if it go beyond, the patient is where no human power can help him. In 1873, there was cholera in Mississippi, Alabama, Georgia, Missouri, Kentucky, Arkansas, Ohio, Illinois, Dakota, Minnesota, and, for the first time, it went to the Rocky Mountains. It was most severe in Mississippi, Kentucky, Illinois, and Eastern Tennessee.

Dr. Hay said that he was glad to have the privilege of hearing the address of Dr. McClellan. Like many other members of the profession, he had been skeptical about the specific character of the epidemic of 1873, until he had had the opportunity, through the kindness of the Sanitary Superintendent of Chicago, (Dr. B. C. Miller), to examine the infected district, and many of the cases thoroughly. The result of this examination was to banish all doubts from his mind. The convictions then formed had been corroborated by the remarks of Dr. McClellan. Dr. Hay thought, moreover, that there was one point in the doctor's remarks which deserved more than a passing notice, and this was the collation of epidemiological with meteorological statistics, as having a most important practical bearing upon our knowledge, and management of epidemics. In fact, the whole subject of epidemiology was a problem of three essential factors—

The first of which was, the specific poison present, *per se;*

The second, atmospheric and telluric conditions favorable to its preservation and transmission; and

Third, a condition of the individual organism favorable to its reception and development.

The absence of any one of these factors prevents the accomplishment of the problem.

In conclusion, Mr. President, I move that the thanks of the Chicago Society of Physicians and Surgeons be tendered to Dr. McClellan for the very interesting and instructive remarks he has kindly given us this evening.

Which being put to vote was carried unanimously.

Dr. I. N. Danforth said, those who have listened attentively, must have been convinced of the existence of specific disease germs; they can be traced, man by man, from well to well, drain to drain. The Government has certainly assigned the right man to the right place.

Dr. McClellan said that he could say one thing, that of all the reports he had yet received, the report of the Board of Health of Chicago was the most comprehensive of any; the facts were clear and to the point, and would give him the greatest assistance. Its microscopical report by Dr. Danforth was the only one he had yet found.

Dr. Owens adverted to the fact that this Society had appointed a committee last year to study the disease. The Sanitary Superintendent was present, and said that the first case of cholera in Chicago, in 1873, occurred on May 24th, at No. 444 Arnold street, in the person of John McFee, a bridge builder, who had been working near Memphis, and left on account of the cholera. When he arrived in Chicago, he had diarrhœa which remained unchecked, and after a week or ten days there were developed choleraic symptoms, which proved fatal.

*Transactions at Regular Meeting, September* 28, 1874.

Dr. J. E. Owens, Vice-President, in the Chair. The Society met, as usual, in one of the parlors of the Grand Pacific Hotel, and, in the absence of Dr. Hyde, Dr. A. Reeves Jackson acted as Secretary.

Dr. Owens gave, briefly, a report of two cases of fracture treated at St. Luke's Hospital, in one of which, that of a colored man thirty-eight years of age, a piece of bone the size of a filbert had been broken off from the left fibula, about two inches above the external malleolus. The case progressed favorably under the usual treatment, by Dupuytren's splint, the fragment of bone speedily united and became incorporated with the long bone itself. The second case, a fracture of the humerus just above the condyles, was of interest, from the fact that it was caused by the throwing of a base-ball.

Dr. H. A. Johnson addressed the Society upon the subject of Pneumatic Aspiration, illustrating the same by exhibiting and explaining the several forms of instruments now employed, exposing at the same time defects, both in theory and construction, of some of the aspirators. The following is a synopsis of his lecture :

In the first place, the question arises as to what is understood by the term aspiration. Is it anything new, either in principle or mechanism ? Is it merely after the fashion of the stomach pump ? Some physicians deny that there is any new principle involved in pneumatic aspiration. In my judgment, Dieulafoy has introduced a new idea, and instrument. It consists in this—the previous production of a vacuum, and then its connection with a cavity containing fluid.

The second question that presents itself, is that of the methods of producing this vacuum. The aspirator of Dieulafoy was then explained. It consists of a glass cylinder in which there is a piston, moved by means of a rack and pinion. The aspirator is enclosed in a metal frame, having a broad and heavy base.

There is attached a graduated scale to measure fluids, and also two stop-cocks. The canules and needles and rubber tubing were also exhibited.

Dr. Johnson showed another form of aspirator, known as the *Aspirateur Potain*, to which, for reasons hereafter to be given, he gave the preference. In referring to an instrument made in Boston, a modification of Potain's, he said that it would be unnecessary to purchase the bottle accompanying it, as the perforated rubber cork, with its double canula, would answer every purpose, and bottles were always easily to be found, in most families.

Thirdly. The next step, is to place the vacuum in relation with the cavity to be emptied. As regards the choice of a needle and canula: there are two kinds of needles in use, each suited to its particular purposes. One of the needles is like the ordinary one, used in hypodermic injections; but this is objectionable in cases where the sharp penetrating point going beyond the wall of the cavity, might injure delicate viscera, as for example the lungs. In many respects the needles and canules, which differ from the needles first described, of the Aspirateur Potain, are greatly to be preferred.

This, the second variety, is really a trochar with a canula, with a branch tube for the attachment of the soft rubber connection. In case of obstruction, the canula can be cleared with a celerity and ease quite impossible to the first variety of needle.

The method of puncture was next explained, and that it was necessary first to determine where you wanted to put the point, and then how deeply the puncture should be made.

In some cases, there are gases as well as fluids in a cavity, and by this means the vacuum of some forms of the aspirator may be disturbed, if not destroyed. The application of pneumatic aspiration has been widely discussed of late years in the medical journals. During the past two years, in which Dr. Johnson has employed this instrument, and in upwards of seventy different cases, he has never seen in a single instance any particular discomfort to the patient, from its use. Among the numerous cases reported, the particulars of a case of an obscure and deep-seated pain in the mammary region, which, upon exploratory puncture an inch and a quarter deep by the aspirator, proved to be an abscess, were most instructive. In another case, that of acute infantile hydrocephalus, he had introduced the needle of the aspirator into the anterior fontanelle to the right of and toward the median line, the point directed obliquely, so as to penetrate the membranes beneath the vessels of the longitudinal sinus. A little fluid was drawn off, with temporary relief to the breathing and circulation. There were no unfavorable symptoms attributable to the operation. The patient, however, subsequently died. In another case, where there was acute effusion in the knee-joint, pneumatic aspiration afforded prompt and decided relief, but in forty-eight hours the effusion had returned. The great assistance to be derived from pneumatic aspiration in differential diagnosis was illustrated by the case of a patient who had been under the care of a homœopathist for so-called uterine trouble with reflex uterine irritation. Upon examination, it was found that the apex of the heart was under the axilla, and that the right chest measured two inches larger than the left. The question then arose as to whether there was a solid morbid growth in the thorax, or a fluid only. Upon introducing the canule of the aspirator into the chest, upwards of two and one-half quarts of muddy serum, not yet quite purulent, were drawn off. The patient for the past year, since that event, has been in perfect health.

The subject of injury to the lungs by aspiration by a penetrating wound of the needle, was then discussed. Several cases of great interest and value were cited, going to show that the danger to the lung in any event would be little or none. A noticeable feature in pneumatic aspiration is, that by this operation there is little probability of introducing from without inwards, any points for future infection of the system. Preference was given to the Aspirateur Potain over that of Dieulafoy, because the vacuum was less uncertain, and is more easily to be maintained. It was not considered to be a point of advantage in Dieulafoy's aspirator, that it might be used for the injection of medicated fluids into cavities, inasmuch as it would be like using an unwashed syringe lately containing unhealthful fluids,—pus, for example,—to throw new and medicated fluids into the body.

Dr. Owens reported a case under the care of Dr. Heydock, in which pneumatic aspiration had been tried upon an effusion in the pericardial sac, of a patient who had pericarditis and endocarditis. At the time of the operation, the pulse was 126, temperature, 101¾°

Fah., and respiration, 66. The precordial dullness extended from the first rib to the upper margin of the sixth; apex impulse indistinctly felt in the fifth intercostal space. Laterally the percussion dullness began at about the middle line of the sternum, and extended around to the left side. The needle of the aspirator was inserted in the fourth intercostal space, one inch and a half from the left margin of the sternum: it was pushed upwards, inwards, and a little backwards. Frothy, dark-colored blood appeared in the receiving bottle, probably because the lung had been pierced. Three-quarters of an hour after this operation, the pulse was 120, and the respiration, 45. Dieulafoy remarks of adults that when the needle has penetrated from 1.17 inch to 2.34 inches, the instrument must meet either the heart or the effusion. The head and shoulders should be elevated with pillows, while operating, and the needle plunged in at the close of an expiration. This patient, a boy fifteen and a half years of age, afterwards died. The autopsy revealed perfect agglutination of the pericardial layers.

Dr. Johnson gave an account of a similar operation performed upon a patient at the County Hospital, and of experiments he had made upon the cadaver. The puncture should be two and one-half inches from the left border of the sternum; if you go nearer to the sternum you must have a longer needle: there were some reasons which inclined him to think favorably of puncturing in the fifth intercostal space, but as his experiments had not yet been concluded, he reserved his opinion upon this point.

Dr. Emmons read a report of a case of uterine interstitial fibroid tumor, treated hypodermically by ergotin; of which the following is an abstract:

Mrs. X, married, age 45, American, the mother of three children, the youngest of whom is eleven years of age, was in good health till the time of her last parturition, which proved to be a difficult one, followed by hour-glass contractions, and permanent impairment of health. Two years ago, a noticeable change had gradually taken place: menstruation had become copious and more prolonged than usual, requiring patient to keep the bed for ten days at a time: she was emaciated, had sciatica and chronic laryngitis. At the time of Dr. Emmons' first visit—December, 1873—in addition to above symptoms, the patient had become so exsanguinous as almost to be in a state of syncope, from constant flooding—there was pain in the uterus and chest—loss of appetite and sleep. There was abdominal tenderness, particularly over the region of the right ovary. The uterus was enlarged, extending up to within two inches of the umbilicus, and a little higher upon the right than left side. Digital examination, per vaginam, revealed anteversion, the os low down against rectum, and dilated to about one-half inch. A metallic sound could be introduced (with strong curve towards anterior wall of abdomen) to a depth of six and one-half inches; a flexible catheter passed an half inch farther. There was a hard tumor commencing at middle of the neck of the uterus, attached to its anterior wall, and extending to the fundus uteri.

Treatment: directed a tonic of iron and quinine, and

| | | |
|---|---|---|
| R. | Ergotin (Bonjeau), - - - - | dr. j. |
| | Aquæ Destil., - - - - | f oz. j. |
| | Glycerini, - - - - - | f dr. j. |

M. Sig. Twelve drops, daily: hypodermically. Improvement was immediate, as shown by the subsidence of pain and hemorrhage. At the end of fifty-nine days, the patient was much better, pulse fuller, appetite better; sleeps well and without anodynes, and able to sit up two hours daily; less abdominal tenderness, but no diminution in size of tumor. The dose of ergotin was increased so as to get about four minims per dose, and continued for three weeks without any particular change in the patient's condition.

The use of Squibb's aqueous solution of ergot, prepared for me so as to get one grain of the drug to the minim, giving twenty minims daily by hypodermic injection over the deltoid, was most satisfactory. Examination twelve days after this change of medicine, revealed the tumor subsided to four and one-half inches below umbilicus. The cavity of uterus measured about five and one-half inches in depth. Patient able to ride out daily; has gained eighteen pounds during the past fortnight, is entirely free from pain; last menstruation normal in quantity, and lasted four days. At another examination in June the fibroid had still farther diminished in size: the uterine cavity measured four inches in depth. Patient complained at this time of a quite free watery discharge from the vagina. A month later, during which time the treatment had been continued, the uterine cavity was found to measure three and one-half inches in depth, watery discharge still present. The treatment was suspended for ten days and then resumed. In August the uterus had regained its normal size, and no tumor remained. The continued daily hypodermic injection, during a period of 156 days, of the twenty minims of a preparation containing Squibb's aqueous solution of ergot, had the effect of gradually reducing the fibroid till hardly a trace of it could be found. There was no particular inconvenience or irritation produced at the points of puncture, especially after laying aside the use of the alcoholic preparation of ergotin.

Dr. Emmons gave some quotations regarding the action of ergot from the results of an experimental investigation by Dr. Warnich, and from recent works of other authorities, such as Drs. Paul Vogt, Ritchie, Handelin, and C. Boldt. *In true interstitial fibroid of the uterus, treated hypodermically with the aqueous solution of ergot, we may expect in the large majority of cases, eminently more satisfactory results than by any other mode of treatment, or by operation.*

Dr. Davis. Why was the medicine not administered by the stomach?

Dr. Emmons replied, because the effect was more satisfactory when given hypodermically; it enters more rapidly into the circulation, and does not disturb the appetite or digestion. He knew of a physician who had given the ordinary tincture of ergot by injection and thereby quickly produced numerous abscesses.

Dr. Merriman said that in a couple of cases under his care there have a quantity of abscesses followed upon the hypodermic use of a solution of ergotine. Afterwards, he gave Squibb's extract of ergot in solution, by the mouth, but it was not so efficient as when given by injection. He had always made the insertion directly over the region of the uterus. Even in treating cases not of interstitial form of fibroids, when giving ergot by the mouth, great benefit has been apparent; the debility is lessened, the menstruation has become more regular in amount and time, the patient feels better even when there has been no diminution in size of the uterine growth.

The Society next discussed the report of the Committee on the Medical Fee Bill, but owing to the lateness of the hour, adjourned, leaving the question of its adoption to a future meeting.

